# Osteoglycin and Sclerostin Imbalance in Hypophosphatasia: Bone-Derived Markers of Mineralization and Systemic Involvement

**DOI:** 10.3390/ijms27157048

**Published:** 2026-08-06

**Authors:** Luis Martínez-Heredia, Clara Toro-Comino, María José Muñoz-Domene, Trinidad González-Cejudo, María Carmen Andreo-López, Victoria Contreras-Bolívar, Cristina García-Fontana, Beatriz García-Fontana, Manuel Muñoz-Torres

**Affiliations:** 1 Instituto de Investigación Biosanitaria de Granada (Ibs. Granada), 18012 Granada, Spain; luismh95@gmail.com (L.M.-H.); ctoro@fibao.es (C.T.-C.); mmunoz@fibao.es (M.J.M.-D.); trinigcej@hotmail.com (T.G.-C.); mcandreo21@gmail.com (M.C.A.-L.); victoriacontreras_87@hotmail.com (V.C.-B.); mmt@mamuto.es (M.M.-T.); 2 CIBER on Frailty and Healthy Aging (CIBERFES), Instituto de Salud Carlos III, 18012 Madrid, Spain; 3 Clinical Analysis Unit, University Hospital Clínico San Cecilio, 18016 Granada, Spain; 4 Endocrinology and Nutrition Unit, University Hospital Clínico San Cecilio, 18016 Granada, Spain; 5 Department of Medicine, University of Granada, 18016 Granada, Spain

**Keywords:** hypophosphatasia, sclerostin, osteoglycin, alkaline phosphatase, systemic diseases

## Abstract

Hypophosphatasia (HPP) is a rare inherited disorder caused by deficient tissue-nonspecific alkaline phosphatase (TNSALP) activity and classically characterized by impaired mineralization processes, although growing evidence suggests broader systemic involvement beyond bone. This cross-sectional study aimed to characterize circulating levels of osteoglycin and sclerostin in patients with HPP and to explore their relationships with TNSALP activity and systemic clinical–biochemical profiles. This cross-sectional study included 25 genetically confirmed HPP patients and 25 age- and sex-matched controls without cardiovascular disease. Circulating osteoglycin and sclerostin were measured by ELISA, and clinical, metabolic, renal, inflammatory, cardiovascular, and bone-related variables were assessed. HPP patients showed lower osteoglycin and higher sclerostin levels compared with controls. Osteoglycin was mainly associated with ALP activity and mineral-related variables, while sclerostin showed broader associations involving glycemic, inflammatory, renal, and cardiovascular domains. In multivariable analyses, osteoglycin was linked to ALP, renal and inflammatory markers, whereas sclerostin was associated with glycemic, mineral, inflammation, and circulatory markers. Overall, osteoglycin variability appeared mainly driven by mineral-related factors, while sclerostin was more influenced by metabolic and inflammatory domains. In conclusion, HPP is associated with an imbalance in circulating bone-derived proteins, characterized by reduced osteoglycin and increased sclerostin, suggesting systemic alterations in bone-related signaling beyond impaired mineralization.

## 1. Introduction

Bone-derived proteins are increasingly recognized as mediators of systemic homeostasis, extending the classical role of bone beyond structural support and mineral storage. In addition to regulating skeletal remodeling and mineralization, several bone-related molecules are involved in the crosstalk between bone and extra-skeletal tissues, including metabolic, renal, immune, and vascular systems [1,2,3]. This concept is particularly relevant in disorders characterized by impaired mineral metabolism, where alterations in bone-derived signaling may contribute to systemic manifestations beyond the skeleton [4,5].

Among these molecules, osteoglycin has attracted growing interest because of its pleiotropic biological functions. Osteoglycin is a small leucine-rich proteoglycan involved in collagen fibrillogenesis, extracellular matrix organization, and bone turnover, processes that are essential for adequate skeletal mineralization [6,7]. Beyond bone tissue, osteoglycin has been related to tissue remodeling, fibrosis, inflammation, and vascular extracellular matrix homeostasis [8]. In addition, experimental and clinical evidence suggests that osteoglycin may participate in energy metabolism by modulating glucose uptake, insulin secretion, and insulin sensitivity [9]. Circulating osteoglycin levels have also been proposed as a potential marker of early renal dysfunction, particularly in patients with type 2 diabetes [10]. Therefore, osteoglycin may represent a bone-derived extracellular matrix protein with potential relevance across skeletal, metabolic, renal, inflammatory, and vascular compartments. Sclerostin, a glycoprotein predominantly secreted by osteocytes and a potent inhibitor of canonical Wnt/β-catenin signaling, is a key regulator of bone formation and skeletal remodeling [11]. However, its biological actions also extend beyond bone. Sclerostin has been detected in vascular tissues, including calcified atherosclerotic plaques, supporting its potential involvement in the bone–vascular axis [12]. Elevated circulating sclerostin levels have been associated with vascular calcification, arterial stiffness, and adverse cardiovascular outcomes in several clinical settings, particularly chronic kidney disease, diabetes, and metabolic disorders [4]. Nevertheless, its vascular role remains controversial, as increased sclerostin may either contribute to vascular dysfunction or represent a compensatory response aimed at limiting excessive mineralization [13,14]. In parallel, sclerostin has emerged as a mediator of osteoimmunological regulation, being involved in the modulation of inflammatory cytokines and chemokines such as IL-6, TNF-α, and MCP-1 [15,16]. This broad biological profile, together with the cardiovascular concerns raised by anti-sclerostin therapies, highlights the need to better understand the systemic actions of sclerostin beyond skeletal tissue [17,18]. Among the wide range of bone-related molecules investigated to date, osteoglycin and sclerostin were selected in the present study because they represent complementary biological pathways involved in skeletal homeostasis. Whereas osteoglycin is mainly related to extracellular matrix organization and mineralization, sclerostin is a key regulator of osteocyte-mediated bone remodeling through inhibition of Wnt/β-catenin signaling. Given their complementary biological functions and reported associations with extra-skeletal processes, these proteins represent suitable candidate biomarkers to investigate the biological heterogeneity of HPP.

Bone-derived proteins have emerged as key regulators not only of skeletal homeostasis but also of cardiovascular physiology and pathology. Increasing evidence supports the existence of a functional bone–vascular axis, in which molecules traditionally associated with bone metabolism show multiple effects on vascular structure, inflammation, endothelial dysfunction, and tissue remodeling [1,2,3]. In this context, several studies have suggested a regulatory role of bone-related proteins in cardiovascular health, highlighting their involvement in extracellular matrix organization, vascular calcification, fibrosis, and atherosclerotic processes [4,5].

Hypophosphatasia (HPP) is a rare, inherited disorder caused by loss-of-function mutations in the *ALPL* gene, which encodes the tissue-nonspecific isoenzyme of alkaline phosphatase (TNSALP) [19]. Deficient TNSALP activity leads to extracellular accumulation of inorganic pyrophosphate (PPi), a well-established inhibitor of hydroxyapatite nucleation and crystal growth, together with an altered balance between inorganic phosphate and pyrophosphate, ultimately resulting in impaired bone and tooth mineralization [20]. The clinical spectrum of HPP is remarkably heterogeneous, ranging from perinatal lethal forms to mild adult-onset presentations, often characterized by musculoskeletal pain, recurrent fractures, chondrocalcinosis, and dental abnormalities [21,22,23]. Although traditionally considered a skeletal disorder, increasing evidence suggests that HPP may have systemic implications beyond bone metabolism, involving mineral homeostasis, metabolic regulation, inflammation, renal involvement, and vascular biology [24].

Altogether, the pleiotropic roles of osteoglycin and sclerostin support the concept that bone-derived proteins participate in multiple physiological processes extending beyond skeletal mineralization, including metabolic, inflammatory, renal, and cardiovascular pathways. In HPP, where TNSALP deficiency profoundly disrupts mineral metabolism, alterations in these circulating proteins may provide valuable insight into the systemic consequences of impaired bone-derived signaling. Accordingly, circulating osteoglycin and sclerostin may serve as candidate biomarkers that reflect alterations in extracellular matrix organization and osteocyte-driven remodeling in HPP. However, their circulating levels and their relationships with ALP activity and clinical, metabolic, renal, inflammatory, cardiovascular, and bone turnover/mineralization-related parameters have not been systematically characterized in this disease.

Therefore, the present study aimed to characterize the circulating profile of osteoglycin and sclerostin in patients with HPP and to determine whether these bone-derived proteins are associated with systemic alterations related to TNSALP deficiency. To this end, we explored their relationships with clinical, metabolic, renal, inflammatory, cardiovascular, and bone turnover/mineralization-related parameters to identify the biological domains contributing to their variability in HPP.

## 2. Results

### 2.1. Clinical, Metabolic, and Mineralization Profile of the Study Population

The baseline clinical and biochemical characteristics of the study population are summarized in Table 1. No significant differences between control and HPP groups were observed regarding age, sex distribution, body mass index, prevalence of type 2 diabetes, dyslipidemia, smoking status, renal function, or estimated cardiovascular risk (SCORE2), indicating a well-balanced study population. Classical metabolic parameters, including fasting glucose, HbA1c, triglycerides, total cholesterol, HDL-C, LDL-C, apolipoprotein B, and eGFR, were also comparable between groups.

As expected, HPP patients exhibited markedly reduced serum ALP activity (*p* < 0.001), accompanied by lower BALP levels (*p* < 0.001) and higher serum phosphorus concentrations (*p* = 0.003), consistent with TNSALP deficiency and altered mineral metabolism. In contrast, classical bone turnover markers were largely comparable between groups, although the P1NP/CTX ratio was slightly lower in HPP patients (*p* = 0.042), with no significant differences in osteocalcin, CTX, or P1NP concentrations. Differences were also observed in selected inflammatory and intestinal-related markers. HPP patients showed higher CRP (*p* = 0.005), eosinophil-to-lymphocyte ratio (ELR) (*p* = 0.043), and fecal calprotectin levels (*p* = 0.029), whereas IL-6 concentrations were lower compared with controls (*p* = 0.047). No significant differences were observed in neutrophil-to-lymphocyte ratio (NLR), lymphocyte-to-monocyte ratio (LMR), or platelet-to-lymphocyte ratio (PLR). As expected, HPP patients exhibited a higher prevalence of disease-related clinical manifestations, including previous fractures (44% vs. 0%, *p* < 0.001) and premature tooth loss (64% vs. 0%, *p* < 0.001), compared with controls.

### 2.2. Circulating Osteoglycin and Sclerostin Levels in HPP Patients

**Figure 1:**
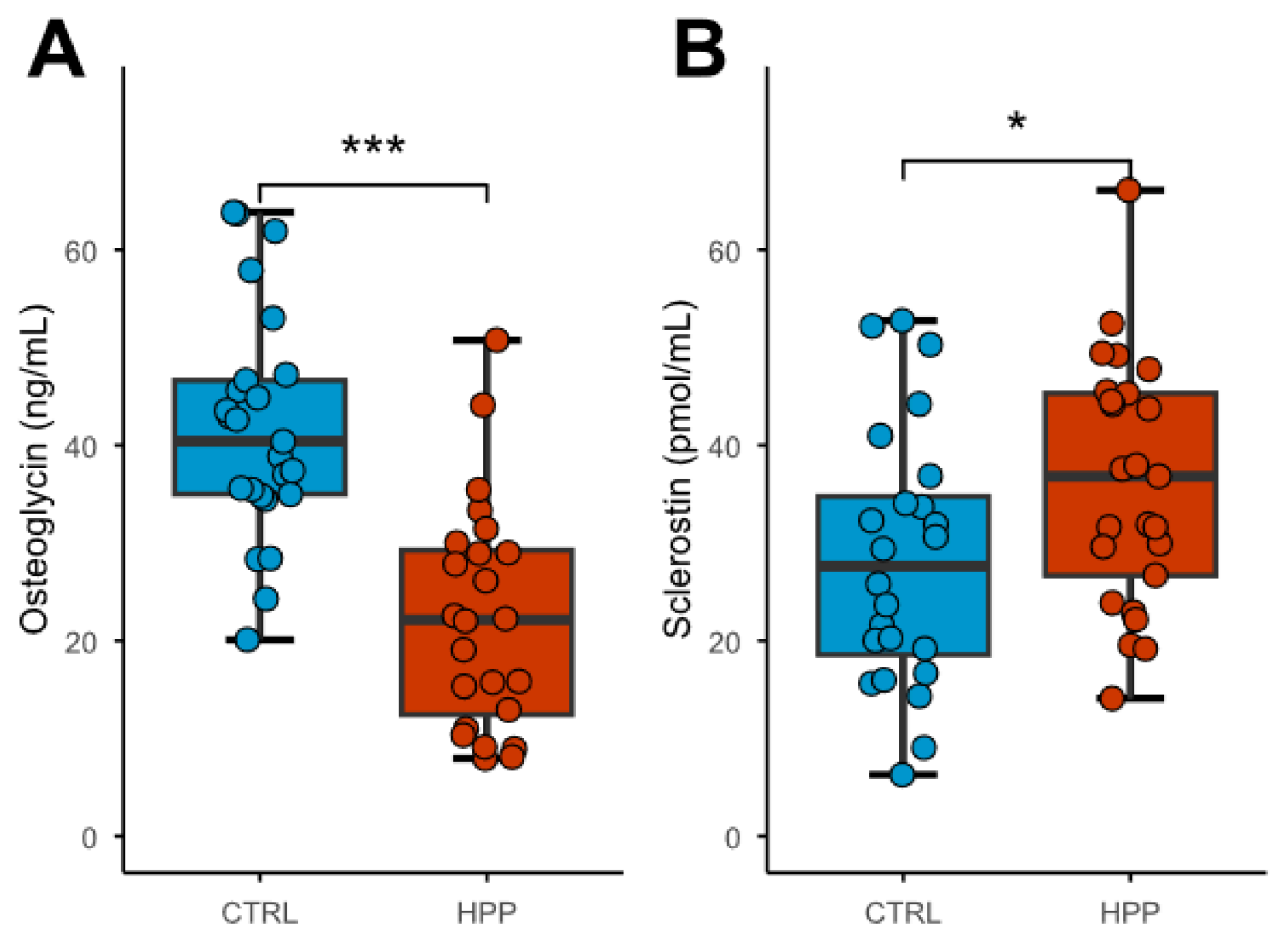
Box plots of serum osteoglycin (**A**) and serum sclerostin (**B**) levels in controls and HPP patients. *p*-values between-group comparisons were calculated using a two-tailed Student’s *t*-test after confirming normal distribution by the Shapiro–Wilk test. *** = *p* < 0.001; * = *p* < 0.05. CTRL: controls; HPP: hypophosphatasia patients.

Comparative analysis of circulating bone-derived biomarkers revealed significant differences between groups (Figure 1). Serum osteoglycin concentrations were markedly reduced in HPP group compared with controls (*p* < 0.001), with a large effect size (Cohen’s d = 1.66). Conversely, circulating sclerostin levels were significantly increased in HPP patients relative to controls (*p* = 0.038), showing a moderate effect size (Cohen’s d = 0.61).

**Figure 2:**
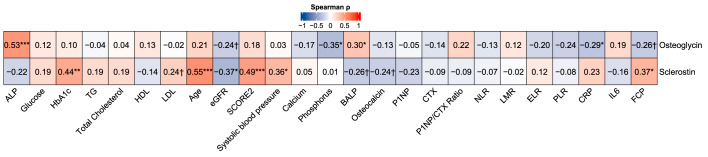
Heatmap plot showing the correlation of osteoglycin and sclerostin with metabolic variables. The correlation coefficients and *p*-values between the different associations were calculated using Spearman’s correlation method. *** = *p* < 0.001; ** = *p* < 0.01; * = *p* < 0.05 and † = *p* < 0.1.

Spearman correlation analysis identified distinct association patterns for osteoglycin and sclerostin across clinical, biochemical, inflammatory, and bone-related parameters (Figure 2). Osteoglycin showed significant positive correlations with serum ALP activity (*p* = 0.53, *p* < 0.001) and BALP concentrations (*p* = 0.30, *p* = 0.042), and inverse correlations with serum phosphorus (*p* = −0.35, *p* = 0.015) and CRP levels (*p* = −0.29, *p* = 0.042). In addition, inverse associations with eGFR, PLR, and fecal calprotectin showed a trend toward significance.

In contrast, sclerostin showed a broader systemic correlation profile. Significant positive correlations were observed with HbA1c (*p* = 0.44, *p* = 0.002), age (*p* = 0.55, *p* < 0.001), SCORE2 (*p* = 0.49, *p* < 0.001), systolic blood pressure (*p* = 0.36, *p* = 0.011), and fecal calprotectin (*p* = 0.37, *p* = 0.011), whereas an inverse correlation was detected with eGFR (*p* = −0.37, *p* = 0.010). Correlations with LDL-C, BALP, and osteocalcin approached statistical significance.

### 2.3. Systemic Determinants of Circulating Osteoglycin and Sclerostin

**Figure 3:**
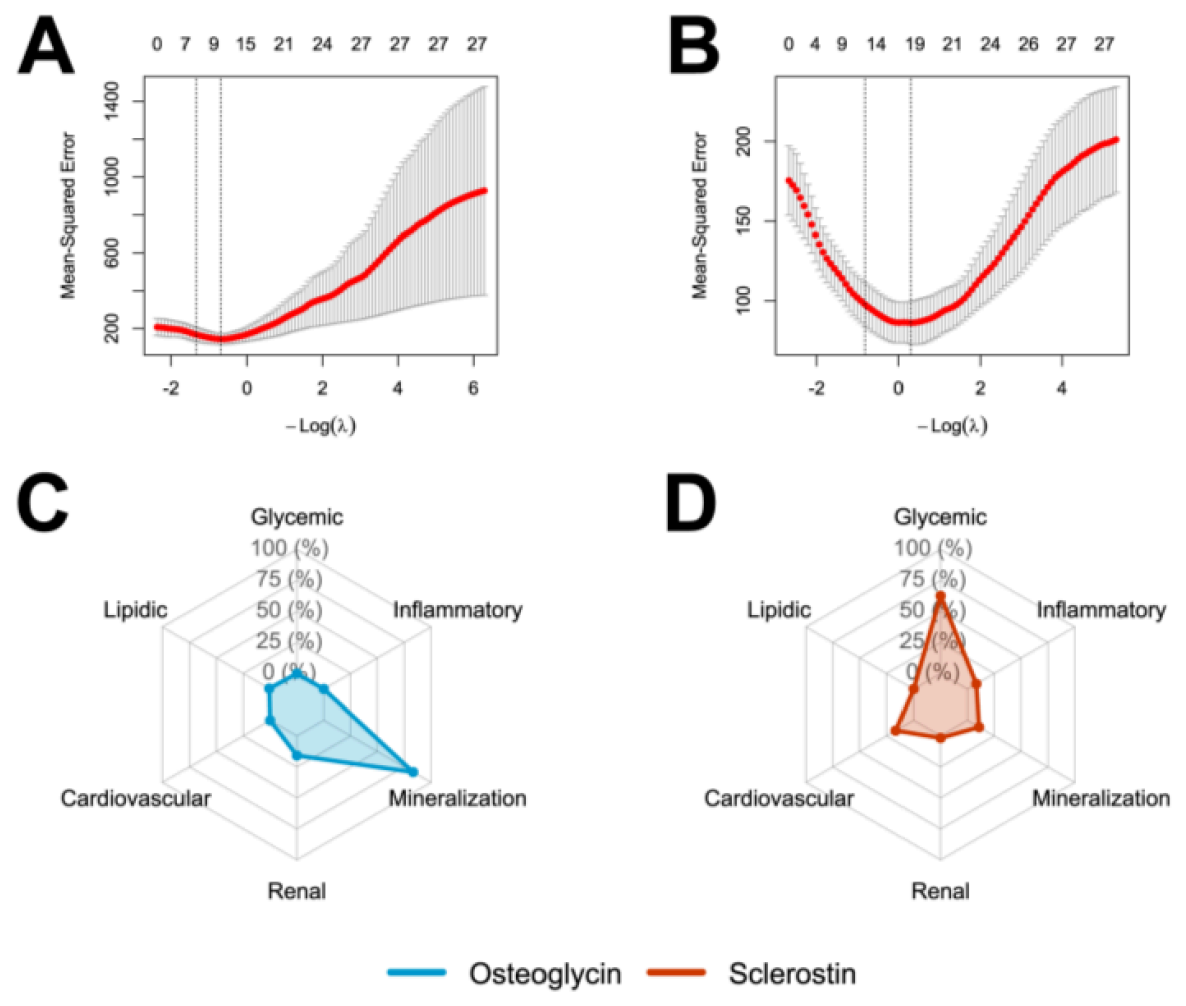
Elastic net regression and functional domain contribution analysis for circulating osteoglycin and sclerostin. (**A**,**B**) Elastic net regression cross-validation curves for osteoglycin (**A**) and sclerostin (**B**). The left dashed vertical line indicates the value of λ that minimizes the cross-validation error (λ_min), whereas the right dashed vertical line represents the one-standard-error criterion (λ_1se), which was used to derive the final parsimonious model. Red dots represent the mean cross-validated error for each λ value, and vertical bars indicate ±1 standard error. (**C**,**D**) Radar plots illustrating the relative contribution of functional domains to the elastic net-derived models of circulating osteoglycin (**C**) and sclerostin (**D**).

To further characterize the systemic determinants of circulating osteoglycin and sclerostin, elastic net regression, domain-level contribution analysis, and multivariable linear regression models were performed. Elastic net regression identified distinct but partially overlapping predictor profiles for both proteins. For osteoglycin, the final model selected ALP, HDL-C, age, eGFR, calcium, P1NP, CTX, and fecal calprotectin as relevant predictors (Figure 3A). In contrast, sclerostin was associated with a broader systemic profile, including ALP, HbA1c, HDL-C, age, eGFR, calcium, phosphorus, BALP, arterial hypertension, sex, NLR, and LMR (Figure 3B).

Unlike elastic net regression, which identifies individual predictors, domain-level contribution analysis provided a biological interpretation by quantifying the relative contribution of broader physiological systems to protein variability. The detailed numerical values of the domain-level contribution analysis are provided in Supplementary Table S1.

Osteoglycin variability was overwhelmingly driven by mineralization-related processes (83.4%), followed by renal factors (15.8%), whereas lipidic, glycemic, cardiovascular, and inflammatory domains contributed minimally (Figure 3C). In contrast, sclerostin showed a dominant glycemic contribution (75.0%), followed by mineralization-related (13.0%) and inflammatory pathways (10.0%), with lower contributions from lipidic and cardiovascular domains (Figure 3D).

In multivariable linear regression, circulating osteoglycin levels were independently associated with ALP activity (β = 0.26, 95% CI 0.14 to 0.38, *p* < 0.001), eGFR (β = −0.55, 95% CI −0.88 to −0.23, *p* = 0.002), and fecal calprotectin (β = −0.01, 95% CI −0.02 to −0.002, *p* = 0.011). The model explained 52.7% of the variance in osteoglycin levels (adjusted R^2^ = 0.53) (Table 2). Regarding sclerostin, it was independently associated with HbA1c (β = 6.87, 95% CI 0.45 to 13.30, *p* = 0.037), calcium (β = 8.58, 95% CI 2.95 to 14.22, *p* = 0.004), phosphorus (β = −11.40, 95% CI −19.07 to −3.72, *p* = 0.005), BALP (β = −1.31, 95% CI −1.87 to −0.74, *p* < 0.001), arterial hypertension (β = 8.49, 95% CI 3.28 to 13.69, *p* = 0.002), and NLR (β = −6.31, 95% CI −9.94 to −2.68, *p* = 0.001). This model showed higher explanatory capacity, accounting for 70% of sclerostin variability (adjusted R^2^ = 0.70) (Table 3).

## 3. Discussion

To our knowledge, this is the first study evaluating circulating osteoglycin and sclerostin levels, two bone-derived proteins with systemic biological actions, in patients with HPP. The main findings of the present study were that HPP patients showed a distinct and divergent profile of these proteins, characterized by markedly reduced osteoglycin and increased sclerostin levels compared with controls. Moreover, both proteins were associated with parameters reflecting TNSALP status, although they showed clearly different systemic signatures: osteoglycin was mainly linked to mineralization-related and renal/inflammatory variables, whereas sclerostin showed a broader profile involving glycemic, mineralization, inflammatory, and cardiovascular-related parameters.

TNSALP plays a central role in skeletal mineralization by regulating the extracellular balance between Pi and PPi. Through the hydrolysis of PPi, a well-established inhibitor of hydroxyapatite nucleation and crystal growth [25], TNSALP contributes to the local availability of inorganic phosphate required for mineral deposition. Consequently, deficient TNSALP activity leads to PPi accumulation and disruption of the Pi/PPi balance, ultimately impairing bone and tooth mineralization [26,27]. Beyond the skeleton, reduced TNSALP activity and increased PPi availability may also influence extracellular matrix mineralization by limiting hydroxyapatite deposition [28], while excessive extracellular PPi may promote calcium pyrophosphate crystal deposition, contributing to manifestations such as chondrocalcinosis and nephrocalcinosis [21,29]. These observations support the concept that TNSALP deficiency has systemic consequences beyond the skeleton and highlight the need for studies integrating vascular phenotyping, direct assessment of ectopic mineralization, and longitudinal cardiovascular outcomes to clarify the cardiovascular implications of alterations in the TNSALP–PPi axis in HPP.

The marked reduction in circulating osteoglycin levels observed in HPP patients may primarily reflect impaired bone matrix organization and reduced osteoblast-related activity secondary to deficient TNSALP function. Osteoglycin is a small leucine-rich proteoglycan [30] involved in collagen fibrillogenesis [31], extracellular matrix organization [8], and bone turnover [32], all essential processes for skeletal mineralization. In line with this biological role, osteoglycin showed positive associations with both ALP activity and BALP concentrations, supporting a close relationship between osteoglycin levels and TNSALP-dependent mineralization. This interpretation was further reinforced by the elastic net and domain-level analyses, in which mineralization-related variables accounted for the largest proportion of osteoglycin variability, and by the multivariable model, where ALP activity remained the strongest independent determinant of circulating osteoglycin levels. Mechanistically, TNSALP has been involved in the regulation of mesenchymal stem cell differentiation and osteoblast proliferation through modulation of Wnt/β-catenin signaling, suggesting that its deficiency may contribute to altered osteoblast function and matrix maturation [33]. In line with this, osteoglycin has been previously described as an extracellular matrix protein involved in bone formation and collagen organization [8].Therefore, reduced osteoglycin concentrations in HPP patients may represent a circulating reflection of altered bone matrix remodeling and impaired mineralization rather than a primarily cardiometabolic signal.

Nevertheless, the independent associations of osteoglycin with eGFR and fecal calprotectin suggest that this protein may also capture extra-skeletal dimensions of HPP. The relationship with eGFR is consistent with previous clinical evidence proposing osteoglycin as a potential marker of early renal dysfunction in patients with type 2 diabetes, where lower concentrations were associated with mild impairment of kidney function [10], suggesting a potential link between this extracellular matrix protein and renal tissue remodeling beyond bone. Additionally, the observed inverse association with fecal calprotectin is of interest, given emerging evidence linking osteoglycin to epithelial repair and tissue regeneration through extracellular matrix remodeling and wound-healing responses [34]. In this context, low-grade intestinal inflammation, reflected by increased fecal calprotectin, could contribute to altered osteoglycin regulation in HPP patients. However, these extra-skeletal associations should be interpreted as exploratory, and further mechanistic studies are required to determine whether osteoglycin acts as a mediator of renal or intestinal involvement or simply reflects broader systemic alterations associated with TNSALP deficiency.

Regarding sclerostin, HPP patients showed significantly higher circulating levels compared with controls. Sclerostin is mainly produced by osteocytes and acts as a key inhibitor of Wnt/β-catenin signaling, although its expression and functions are not restricted to the osteocyte or skeletal tissue [11,35]. By contrast, whereas osteoglycin variability was mainly explained by mineralization-related variables, sclerostin displayed a broader systemic profile. This is consistent with previous evidence showing that circulating sclerostin levels are influenced by age, sex, body composition, bone mineral content, renal function, glucose metabolism, and cardiovascular risk-related factors [4,5,36,37,38]. In our cohort, correlation analyses showed significant associations with age, HbA1c, systolic blood pressure, SCORE2, eGFR, and fecal calprotectin, while elastic net regression selected variables related to glycemic, mineralization, cardiovascular, renal, and inflammatory domains. However, glucose and HbA1c levels were comparable between HPP patients and controls, suggesting that the increase in sclerostin observed in HPP patients is unlikely to be explained solely by differences in glycemic status. Rather, these findings suggest that circulating sclerostin may be influenced by glycemic regulation at an individual level, while the between-group increase observed in HPP may be more closely related to altered mineralization and inflammatory-related pathways. Overall, sclerostin in HPP patients appears to reflect a complex systemic signature integrating metabolic, mineral, renal, inflammatory, and cardiovascular-related signals rather than a purely skeletal or vascular phenotype.

In this context, TNSALP deficiency may be functionally linked to altered Wnt/b-catenin signaling, given the close interplay between mineral metabolism and osteocyte-derived regulatory pathways. In the present study, sclerostin was independently and inversely associated with BALP, supporting a link between increased osteocyte-derived inhibitory signaling and reduced bone-forming activity. Alternatively, elevated sclerostin could also contribute to limiting inappropriate osteogenic signaling in extra-skeletal tissues, although whether this response is protective or maladaptive cannot be determined from the present data. Therefore, the relationship between TNSALP deficiency, bone turnover, ectopic mineralization, and sclerostin regulation is likely to be bidirectional and tissue-dependent.

The present findings also indicate alterations in inflammatory-related markers in HPP patients, with a particularly relevant intestinal component. HPP patients showed higher CRP, ELR, and fecal calprotectin levels compared with controls, whereas circulating IL-6 concentrations were lower. This apparently discordant pattern suggests that inflammatory alterations in HPP may not reflect a uniform systemic inflammatory response, but rather a more complex and potentially compartmentalized pattern involving different biological compartments, with fecal calprotectin supporting the presence of intestinal inflammatory activity. In this context, the positive correlation between circulating sclerostin and fecal calprotectin, together with the contribution of inflammatory-related variables to the elastic net-derived sclerostin model, supports a potential association between sclerostin regulation and intestinal inflammatory or tissue-remodeling processes in HPP. This interpretation is biologically plausible, as sclerostin has been involved in osteoimmunological regulation, including the modulation of inflammatory cytokines and chemokines such as interleukin-6 (IL-6), tumor necrosis factor-α (TNF-α), and monocyte chemoattractant protein-1 (MCP-1) [16,39]. This immunomodulatory effect has been suggested to involve, at least partially, structural regions within sclerostin, particularly its loop 2 and loop 3 domains, which may contribute to interactions with signaling partners involved in inflammatory regulation [16]. However, the lower IL-6 levels observed in HPP patients should be interpreted cautiously, and the present cross-sectional design does not allow determining whether increased sclerostin contributes to inflammatory regulation or instead reflects broader systemic alterations associated with TNSALP deficiency.

The distinct biological signatures observed for osteoglycin and sclerostin suggest that these proteins may provide complementary information for the biological characterization and stratification of HPP patients. The marked reduction in osteoglycin levels and its association with ALP activity, BALP concentrations, and mineralization-related variables support its potential role as a marker of altered bone matrix organization and mineralization status. Conversely, the increased sclerostin levels and their broader associations with metabolic, inflammatory, renal, cardiovascular, and mineralization-related parameters suggest that sclerostin may reflect the systemic component of HPP beyond skeletal alterations. Further studies in larger longitudinal HPP cohorts, as well as comparisons with more prevalent metabolic bone diseases such as osteoporosis, will be required to determine the specificity, prognostic value, and potential utility of these proteins for patient stratification, disease monitoring, and clinical decision-making.

The present study has some limitations: First, the relatively small sample size, inherent to the rarity of HPP, together with the cross-sectional design, may limit the statistical power and robustness of multivariable analyses and precludes establishing causal relationships between TNSALP deficiency and circulating osteoglycin or sclerostin levels. Second, the presence of cardiometabolic comorbidities, including type 2 diabetes mellitus, and the use of concomitant medications may have introduced residual confounding. Although these variables were recorded and the prevalence of the corresponding clinical conditions did not differ significantly between groups, the limited sample size precluded a comprehensive assessment of the independent effects of each comorbidity and treatment. Third, parathyroid hormone and 25(OH)D levels were not available in the control group, limiting a more comprehensive evaluation of calcium–phosphate regulatory pathways that may influence circulating osteoglycin and sclerostin levels in the present cohort. Fourth, the lack of detailed skeletal and clinical phenotyping, including bone mineral density, radiographic assessments, standardized severity scores, and systematically collected information on age at clinical onset, represents an additional limitation. Since osteoglycin and sclerostin are involved in bone matrix organization and remodeling, future studies integrating imaging and clinical severity measures are needed to determine their relationship with specific skeletal phenotypes in HPP. Fifth, FCP was assessed without detailed gastrointestinal phenotyping. As FCP is a non-specific marker, its elevation alone cannot establish the presence or origin of intestinal inflammatory activity in this cohort. Accordingly, the observed relationship between FCP and circulating sclerostin should be considered exploratory and cannot demonstrate that intestinal inflammation regulates sclerostin or that sclerostin contributes to intestinal inflammatory processes. Finally, as patients were recruited from a single center, the characteristics of the study population may have influenced the observed findings. It is important to note that, although the study included a broad systemic characterization, the absence of mechanistic experiments and longitudinal follow-up prevents determining whether these proteins actively contribute to extra-skeletal manifestations or simply reflect systemic alterations associated with HPP. Thus, further larger and longitudinal studies are needed to evaluate the potential diagnostic applicability and clinical utility of sclerostin and osteoglycin as biomarkers. Nevertheless, this study also has relevant strengths, including the use of a genetically confirmed HPP cohort, age- and sex-matched controls, and the integration of clinical, metabolic, renal, cardiovascular, inflammatory, and bone turnover/mineralization-related variables. In addition, the combination of elastic net regression, domain-level contribution analysis, and multivariable modeling allowed a comprehensive exploratory assessment of the systemic profiles associated with osteoglycin and sclerostin in HPP.

## 4. Materialsand Methods

### 4.1. Study Population

This study included 50 adult participants: 25 patients diagnosed with adult HPP confirmed by *ALPL* gene sequencing and 25 age- and sex-matched controls. All HPP patients were recruited from the Andalusian region at the University Hospital Clínico San Cecilio of Granada. Genetic analysis confirmed the presence of heterozygous *ALPL* variants in all patients. The age- and sex-matched controls showed serum ALP activity within the reference range (>40 IU/L) and had no clinical evidence suggestive of HPP. None of the included HPP patients were receiving asfotase alfa treatment at the time of study inclusion. A previous history or clinical evidence of cardiovascular disease (CVD) was considered an exclusion criterion for both groups.

### 4.2. Clinical Evaluation of Study Population

Body mass Index was calculated by the Quetelet formula (weight (Kg)/height (m^2^)). Dyslipidemia was characterized by serum levels of total cholesterol >200 mg/dL, low-density lipoprotein cholesterol (LDL-C) >130 mg/dL, high-density lipoprotein cholesterol (HDL-C) < 40, triglycerides >150 mg/dL and/or current treatment with lipid-lowering drugs. Hypertension was defined as systolic blood pressure ≥140 mmHg and/or diastolic blood pressure ≥90 mmHg, or current antihypertensive treatment. Smoking status was recorded, and cardiovascular risk was estimated using SCORE2 for participants younger than 70 years or SCORE2-OP for participants aged 70 years or older. Prevalent fractures, premature tooth loss and incidence of type 2 diabetes mellitus were recorded based on clinical history. All diabetic participants included in the study were receiving metformin treatment.

### 4.3. Biochemical Measures of the Study Population

Samples of venous blood were collected in the morning after fasting overnight. Serum samples were stored at −80 °C until analysis. Glucose, glycated hemoglobin (HbA1c), triglycerides, total cholesterol, HDL-C, LDL-C, phosphorus, calcium, and C-reactive protein (CRP) were measured in the hospital following standard automated laboratory techniques. The estimated glomerular filtration rate (eGFR) was calculated following the Chronic Kidney Disease Epidemiology Collaboration equation (CKD-EPI).

Complete blood count parameters, including platelet, lymphocyte, monocyte, neutrophil, and eosinophil counts, were used to calculate the NLR, LMR, ELR, and PLR.

The serum alkaline phosphatase activity (ALP) was determined by the colorimetric method using the AU5800 analyzer (Beckman Coulter, Brea, CA, USA). The bone-specific isoform of alkaline phosphatase (BALP) was also measured in an AU5800 analyzer (Beckman Coulter) using an immunoenzymatic assay. Total osteocalcin was determined by chemiluminescent immunoassay (CLIA) (N-Mid Osteocalcin; Immunodiagnostic Systems iSYS automated analyzer)(IDS, Boldon, UK). The procollagen type 1 N-terminal propeptide (P1NP) and serum carboxy-terminal crosslinked telopeptide of type I collagen (CTX) were determined by electrochemiluminescence immune assay (ECLIA) (Roche Diagnostics, Basel, Switzerland). Circulating osteoglycin and sclerostin levels were determined by the enzyme-linked immunosorbent assay (ELISA) method, following the manufacturer’s protocol (Cloud Clone Corp., Katy, TX, USA and Biomedica Gruppe, Vienna, Austria, respectively). Precision testing was performed by the determination of intra-assay and inter-assay variations for each ELISA (5% and 1% for sclerostin; 10% and 12% for osteoglycin).

Fecal calprotectin (FCP) and interleukin 6 (IL6) were determined by ELISA in the Clinical Analysis Unit of the University Hospital Clínico San Cecilio of Granada.

### 4.4. Statistical Analysis

Analyses and figures were performed in RStudio version 4.5.1. Normality of the variables was assessed using the Shapiro–Wilk test. Between-group comparisons were performed using two-tailed Student’s t-test for normally distributed variables and the Mann–Whitney U test for non-normally distributed variables. Two-tailed tests were selected because no predefined directional hypothesis regarding circulating osteoglycin or sclerostin levels in HPP patients was established. Effect sizes were estimated using Cohen’s d for normally distributed variables and for non-normally distributed variables Cliff’s delta was used. Fisher’s exact test was used to compare the categorical variables between the groups. Correlations between continuous variables were performed by Spearman’s method. Multiple linear regression models were used to determine the variables independently associated with osteoglycin and sclerostin, including categorical variables. Prior to multivariable modeling, an elastic net regression approach was applied for variable selection to identify the most relevant predictors among clinical, biochemical, cardiovascular, and bone turnover-related parameters. All predictor variables were standardized before elastic net analysis. The optimal mixing parameter (alpha) was selected based on cross-validated error minimization, and the final model was derived using the one-standard-error (λ1se) criterion. This procedure allowed for the selection of the most stable subset of predictors while accounting for multicollinearity and overfitting. To explore the relative contribution of broader biological systems, predictors were grouped into predefined functional domains (glycemic, lipidic, cardiovascular, renal, mineralization, and inflammatory). For each domain, the absolute elastic net coefficients of included variables were summed and normalized by the number of variables assigned to that domain to avoid overrepresentation of domains containing more predictors. The resulting domain scores were then normalized to express each domain as a percentage of the total contribution across all domains. Radar plots were used to visualize the relative contribution of each biological domain to osteoglycin and sclerostin variability. Data from the final multivariable models were presented as regression coefficients (β), 95% confidence intervals (CI), and *p*-values. Multicollinearity among independent variables was assessed using the variance inflation factor (VIF), considering the absence of significant collinearity when values were below 5. Model performance was evaluated using the adjusted coefficient of determination (adjusted R^2^).

## 5. Conclusions

In conclusion, this study provides the first evidence that HPP patients present a divergent circulating profile of two bone-derived proteins with systemic biological actions, characterized by reduced osteoglycin and increased sclerostin levels. Osteoglycin was mainly associated with TNSALP-dependent mineralization and extracellular matrix-related processes, whereas sclerostin displayed a broader systemic signature involving glycemic, mineral, inflammatory, renal, and cardiovascular-related signals. These findings extend current knowledge of HPP beyond defective skeletal mineralization and suggest that TNSALP deficiency may be linked to broader alterations in bone-derived molecular signaling. Although the clinical relevance of these changes remains to be established, this work opens new perspectives for investigating whether osteoglycin and sclerostin participate in the systemic alterations observed in HPP and whether they may serve as exploratory biomarkers contributing to future strategies for systemic risk stratification and individualized preventive approaches in future longitudinal studies.

## Figures and Tables

**Table 1:** Comparison of baseline characteristics between control and HPP groups.

Baseline Characteristics	CTRL (n = 25)	HPP (n = 25)	*p*-Value (Effect Size)
Men/Women (n)	9/16	9/16	1.000
Fractures (%)	0	44	<0.001
Tooth Loss (%)	0	64	<0.001
T2D (%)	8	16	0.663
Dyslipidemia (%)	52	44	0.777
Smoker (%)	20	20	1.000
Hypertension (%)	40	40	1.000
CVD Risk (%)	28	32	1.000
SCORE2 (%)	2.80 [1.50–5.70]	2.90 [1.00–4.50]	0.634
Age (>18 years)	50.48 (±15.87)	50.24 (±16.71)	0.959
BMI (Kg/m^2^)	25.14 (±4.03)	26.69 (±4.44)	0.740
ALP (43–120 IU/L)	80.00 [60.00–90.00]	25.00 [21.00–30.00]	<0.001 *** (d: 3.31)
Glucose (70–100 mg/dL)	92.00 [83.00–95.00]	84.00 [80.00–93.00]	0.252
HbA1c (<5.7%)	5.50 [5.40–5.60]	5.50 [5.10–5.82]	0.832
TG (<150 mg/dL)	78.00 [62.00–137.00]	87.00 [71.00–122.00]	0.580
Total Cholesterol (<200 mg/dL)	201.24 (±34.21)	194.44 (±6.30)	0.499
HDL-c (>40 mg/dL)	57.04 (±11.35)	58.64 (±15.07)	0.674
LDL-c (<130 mg/dL)	124.26 (±28.25)	118.32 (±30.04)	0.450
ApoB (<109 mg/dL)	95.76 (±19.31)	95.83 (±22.61)	0.990
Sclerostin (pmol/L)	28.24 (±13.22)	36.15 (±12.6)	0.038 * (d: 0.612)
Osteoglycin (ng/mL)	41.76 (±11.65)	22.42 (±11.65)	<0.001 *** (d: 1.66)
eGFR (≥90 mL/min/1.73 m^2^)	90.00 [87.00–90.00]	90.00 [81.12–90.00]	0.673
Systolic blood pressure (90–120 mmHg)	120.00 [120.00–129.00]	123.00 [119.00–135.00]	0.861
Calcium (8.6–10.3 mg/dL)	9.53 (±0.38)	9.61 (±0.48)	0.497
Phosphorus (2.5–4.5 mg/dL)	3.18 (±0.44)	3.59 (0.50)	0.003 ** (d: 0.877)
Bone Alkaline Phosphatase (5.7–24.7 ng/mL)	11.60 [7.88–15.85]	4.32 [3.05–6.58]	<0.001 *** (δ: 0.799)
Osteocalcin (10.4–45.6 ng/mL)	20.40 [18.70–22.20]	20.2 [16.90–22.75]	0.648
CTX (0.112–1.008 ng/mL)	0.38 [0.30–0.56]	0.40 [0.27–0.59]	0.810
P1NP (16–96 ng/mL)	53.90 [40.00–67.80]	39.40 [31.00–58.80]	0.126
P1NP/CTX	126.51 [106.62–169.59]	107.00 [82.71–123.08]	0.042 * (δ: 0.340)
NLR	1.79 [1.48–2.11]	1.78 [1.29–2.50]	0.715
LMR	4.48 [3.40–4.98]	3.60 [2.89–5.37]	0.289
ELR	0.06 [0.04–0.10]	0.10 [0.06–0.13]	0.043 * (δ: 0.334)
PLR	114.84 (±32.12)	120.41 (±34.43)	0.558
CRP (<3 mg/L)	1.40 [0.70–2.40]	2.90 [1.53–4.93]	0.005 ** (δ: 0.467)
IL6 (<5.9 mg/L)	3.09 [2.40–3.80]	2.00 [1.60–2.59]	0.047 * (δ: 0.400)
FCP (>50 ug/g)	39.20 [1.00–214.00]	98.00 [50.25–205.75]	0.029 * (δ: 0.371)

CVD: cardiovascular disease; ALP: serum alkaline phosphatase; HbA1c: glycated haemoglobin; TG: triglycerides; HDL-C: high-density lipoprotein cholesterol; LDL-C: low-density lipoprotein cholesterol; ApoB: apolipoprotein B; eGFR: estimated glomerular filtration rate. BALP: Bone alkaline phosphatase isoform; CTX: carboxy-terminal crosslinked telopeptide of type I collagen; P1NP: procollagen type 1 N-terminal propeptide. NLR: Neutrophil-to-lymphocyte Ratio, LMR: lymphocyte-to-monocyte ratio; ELR: eosinophil-to-lymphocyte ratio; PLR: platelet-to-lymphocyte ratio; CRP: C-reactive protein; IL6: interleukin-6; FCP: fecal calprotectin. Continuous variables are expressed as mean (±SD) for normal distributed variables and median with IQR (Q1–Q3) for non-normally distributed variables. Between-group comparisons were performed using two-tailed Student’s t-test for normally distributed variables and the Mann–Whitney U test for non-normally distributed variables. *** = *p* < 0.001; ** = *p* < 0.01; * = *p* < 0.05.. Effect sizes were calculated as Cohen´s d for significant parametric variables and as Cliff´s delta (δ) for significant non-parametric variables.

**Table 2:** Osteoglycin multivariable linear regression.

	β	CI 95%	*p*-Value	VIF
ALP	0.26	0.14, 0.38	<0.001 **	1.28
HDL	0.17	−0.08, 0.43	0.179	1.24
Age	0.056	−0.20, 0.31	0.656	1.74
eGFR	−0.56	−0.88, −0.23	0.002 **	1.77
Calcium	−4.99	−12.94, 2.95	0.210	1.28
P1NP	−0.07	−0.27, 0.13	0.484	2.58
CTX	−13.68	−29.95, 2.60	0.097	1.83
FCP	−0.01	−0.02, −0.002	0.011 *	1.57
Adjusted R^2^	0.53			

ALP: serum alkaline phosphatase; HDL-C: high-density lipoprotein cholesterol; eGFR: estimated glomerular filtration rate; P1NP: procollagen type 1 N-terminal propeptide; CTX: carboxy-terminal crosslinked telopeptide of type I collagen; FCP: fecal calprotectin; CI: confidence interval; VIF: variance inflation factor. Data are presented as regression coefficients (β), 95% confidence intervals, *p*-values, and variance inflation factors.** = *p* < 0.01; * = *p* < 0.05.

**Table 3:** Sclerostin multivariable linear regression.

	β	CI 95%	*p*-Value	VIF
Hb1Ac	6.87	0.45, 13.30	0.037 *	1.81
HDL	−0.14	−0.36, 0.08	0.206	1.79
Age	0.08	−0.12, 0.27	0.437	2.04
eGFR	−0.20	−0.41, 0.02	0.069	1.42
Calcium	8.58	2.95, 14.22	0.004 **	1.24
Phosphorus	−11.40	−19.07, −3.72	0.005 **	2.56
BALP	−1.31	−1.87, −0.74	<0.001 ***	1.99
Hypertension	8.49	3.28, 13.69	0.002 **	1.34
Sex	1.40	−6.27, 9.07	0.712	2.83
NLR	−6.31	−9.94, −2.68	0.001 **	1.57
LMR	−1.21	−3.46, 1.05	0.284	2.14
Adjusted R^2^	0.70			

HbA1c: glycated hemoglobin; HDL-C: high-density lipoprotein cholesterol; eGFR: estimated glomerular filtration rate; BALP: bone alkaline phosphatase isoform; NLR: neutrophil-to-lymphocyte ratio; LMR: lymphocyte-to-monocyte ratio; CI: confidence interval; VIF: variance inflation factor. Data are presented as regression coefficients (β), 95% confidence intervals, *p*-values, and variance inflation factors. *** = *p* < 0.001; ** = *p* < 0.01; * = *p* < 0.05.

## Data Availability

The data underlying this study are not publicly available due to privacy issues. The data is available by request from the corresponding author.

## Supplementary Materials

The following supporting information can be downloaded at https://www.mdpi.com/article/10.3390/ijms27157048/s1.

## Abbreviations

The following abbreviations are used in this manuscript:

ALP	alkaline phosphatase.
ALPL	alkaline phosphatase, liver/bone/kidney gene.
ApoB	apolipoprotein B.
BALP	bone alkaline phosphatase.
BMI	body mass index.
CI	confidence interval.
CKD-EPI	Chronic Kidney Disease Epidemiology Collaboration.
CLIA	chemiluminescent immunoassay.
CRP	C-reactive protein.
CTX	carboxy-terminal crosslinked telopeptide of type I collagen.
CTRL	control group.
CVD	cardiovascular disease.
ECLIA	electrochemiluminescence immunoassay.
ECM	extracellular matrix.
eGFR	estimated glomerular filtration rate.
ELR	eosinophil-to-lymphocyte ratio.
FCP	fecal calprotectin.
HbA1c	glycated hemoglobin.
HDL-C	high-density lipoprotein cholesterol.
HPP	hypophosphatasia.
IL-6	interleukin-6.
IQR	interquartile range.
LDL-C	low-density lipoprotein cholesterol.
LMR	lymphocyte-to-monocyte ratio.
MCP-1	monocyte chemoattractant protein-1.
NLR	neutrophil-to-lymphocyte ratio.
P1NP	procollagen type I N-terminal propeptide.
Pi	inorganic phosphate.
PLR	platelet-to-lymphocyte ratio.
PPi	inorganic pyrophosphate.
SCORE2	Systematic Coronary Risk Evaluation 2.
SCORE2-OP	Systematic Coronary Risk Evaluation 2-Older Persons.
T2D	type 2 diabetes.
TG	triglycerides.
TNSALP	tissue-nonspecific alkaline phosphatase.
TNF-α	tumor necrosis factor-alpha.
VIF	variance inflation factor.
