# Supplementary material for: Osteoglycin and Sclerostin Imbalance in Hypophosphatasia: Bone-Derived Markers of Mineralization and Systemic Involvement
Source: Int J Mol Sci. 2026 Aug 6;27(15):7048. doi: 10.3390/ijms27157048 (PMC13467457; doi:10.3390/ijms27157048)
Supplement: Supplementary file 1 [file ijms-27-07048-s001.zip › ijms-4431389-supplementary.pdf]

Supplementary Table S1. Detailed normalized contribution values of individual domains for Sclerostin and Osteoglycin

Biological domain	Osteoglycin contribution	Sclerostin contribution
Glycemic	0	62.7
Lipidic	0.8	0.1
Cardiovascular	0	16.4
Renal	15.8	1.6
Mineralization	83.4	10.9
Inflammatory	0.1	8.3
